# SARS-CoV-2 Seroprevalence and Drug Use in Trauma Patients from Six Sites in the United States

**DOI:** 10.1101/2021.08.10.21261849

**Published:** 2021-08-11

**Authors:** Tran B. Ngo, Maria Karkanitsa, Kenneth M. Adusei, Lindsey A. Graham, Emily E. Ricotta, Jenna R. Darrah, Richard D. Blomberg, Jacquelyn Spathies, Kyle J. Pauly, Carleen Klumpp-Thomas, Jameson Travers, Jennifer Mehalko, Matthew Drew, Matthew D Hall, Matthew J Memoli, Dominic Esposito, Rosemary A. Kozar, Christopher Griggs, Kyle W. Cunningham, Carl I. Schulman, Marie Crandall, Mark Neavyn, Jon D. Dorfman, Jeffrey T. Lai, Jennifer M. Whitehill, Kavita M. Babu, Nicholas M. Mohr, Jon Van Heukelom, James C. Fell, Whit Rooke, Heather Kalish, F. Dennis Thomas, Kaitlyn Sadtler

**Affiliations:** 1 Section on Immuno-Engineering. National Institute of Biomedical Imaging and Bioengineering, National Institutes of Health, Bethesda MD 20894; 2 Dunlap and Associates, Inc., Stamford CT 06906; 3 Epidemiology and Population Studies Unit, National Institute of Allergy and Infectious Diseases, National Institutes of Health, Bethesda MD 20894; 4 Bioengineering and Physical Sciences Shared Resource, National Institute for Biomedical Imaging and Bioengineering, National Institutes of Health, Bethesda MD 20894; 5 National Center for Advancing Translational Sciences, National Institutes of Health, Rockville MD 20852; 6 Protein Expression Laboratory, Frederick National Laboratory for Cancer Research, Frederick MD 21702; 7 Clinical Studies Unit, Laboratory of Infectious Diseases, National Institute for Allergy and Infectious Diseases, National Institutes of Health, Bethesda MD 20894; 8 Shock Trauma Center, University of Maryland School of Medicine, Baltimore MD 21201; 9 Department of Emergency Medicine, Atrium Health’s Carolinas Medical Center, Charlotte NC 28203; 10 Division of Acute Care Surgery, Atrium Health’s Carolinas Medical Center, Charlotte NC 28203; 11 University of Miami Miller School of Medicine, Miami FL 33136; 12 Department of Surgery, University of Florida College of Medicine, Jacksonville FL 33209; 13 Department of Emergency Medicine, University of Massachusetts Medical School, Worcester MA 01655; 14 Maine Medical Center, Department of Emergency Medicine, Tufts University School of Medicine, Portland ME 04102; 15 Division of Medical Toxicology, Department of Emergency Medicine, University of Massachusetts Medical School, Worcester MA 01655; 16 Department of Health Promotion and Policy, University of Massachusetts Amherst, Amherst MA 01003; 17 Division of Medical Toxicology, Department of Emergency Medicine, University of Massachusetts Medical School, Worcester MA 01655; 18 Department of Emergency Medicine, Anesthesia Critical Care, and Epidemiology, University of Iowa Health Care, Iowa City IA 52242; 19 Department of Emergency Medicine, University of Iowa Health Care, Iowa City IA 52242; 20 NORC at the University of Chicago, Bethesda, MD 20814; 21 KIYATEC, Inc., Greenville SC 29605

## Abstract

In comparison to the general patient population, trauma patients show higher level detections of bloodborne infectious diseases, such as Hepatitis and Human Immunodeficiency Virus. In comparison to bloodborne pathogens, the prevalence of respiratory infections such as SARS-CoV-2 and how that relates with other variables, such as drug usage and trauma type, is currently unknown in trauma populations. Here, we evaluated SARS-CoV-2 seropositivity and antibody isotype profile in 2,542 trauma patients from six Level-1 trauma centers between April and October of 2020 during the first wave of the COVID-19 pandemic. We found that the seroprevalence in trauma victims 18–44 years old (9.79%, 95% confidence interval/CI: 8.33 – 11.47) was much higher in comparison to older patients (45–69 years old: 6.03%, 4.59–5.88; 70+ years old: 4.33%, 2.54 – 7.20). Black/African American (9.54%, 7.77 – 11.65) and Hispanic/Latino patients (14.95%, 11.80 – 18.75) also had higher seroprevalence in comparison, respectively, to White (5.72%, 4.62 – 7.05) and Non-Latino patients (6.55%, 5.57 – 7.69). More than half (55.54%) of those tested for drug toxicology had at least one drug present in their system. Those that tested positive for narcotics or sedatives had a significant negative correlation with seropositivity, while those on anti-depressants trended positive. These findings represent an important consideration for both the patients and first responders that treat trauma patients facing potential risk of respiratory infectious diseases like SARS-CoV-2.

## INTRODUCTION

The Coronavirus Disease 2019 (COVID-19) pandemic has been a daunting medical challenge for scientists, clinicians, and healthcare professionals due to the ability of the SARS-CoV-2 virus to spread quickly and, frequently, undetected. Currently, there are over 200 million confirmed cases of COVID-19 globally, with the United States accounting for almost 18 % of these cases^1^. The U.S. prevalence and disparities of SARS-CoV-2 infection have been documented in different demographics and regional areas^2–7^. However, this statistic undercounts pre-symptomatic and asymptomatic patients, both of whom can transmit SARS-CoV-2^8^; hence, the number of people spreading SARS-CoV-2 at any given time is difficult to determine.

Furthermore, there is limited information regarding the prevalence of COVID-19 in patients admitted to hospitals due to trauma. Previous studies indicate that trauma victims have a higher prevalence of certain viral infections, such as Human Immunodeficiency Virus (HIV). In 2018, researchers showed that 1.1% of 1217 individuals in a trauma cohort tested positive for HIV, which was more than three times the national prevalence estimated by the Centers for Disease Control and Prevention (CDC) of the U.S. general population (0.37% or 1.2 million HIV positive cases)^9, 10^. Other viral infection prevalence is also higher in trauma patients than the national average. In a study analyzing positivity of bloodborne viruses Hepatitis B/C and HIV, 75% of patients who tested positive were undiagnosed for these diseases prior to enrollment^11^. Injury severity, another pre-hospital factor, has been shown to be an independent predictor of ventilator-associated pneumonia causing complications in trauma population^12^. Overall, trauma patients require direct and intensive care from many health care providers including the first responders (e.g., emergency medical services/EMS, law enforcement), primary trauma team (e.g., treating medical staff in trauma centers), and specialists (e.g. respiratory, physical, and occupational therapy)^13^.

With a high community transmission rate of SARS-CoV-2 virus along with many variant lineages of concern, first responders and health care workers could be facing a much higher risk of exposure to viral infection than previously expected when treating trauma patients. As reported, COVID-19 related fatality risks were the single highest cause of officer line-of-duty deaths^14, 15^. EMS providers, who have been operating on the far-forward front lines of the pandemic in 2020, had more cases of severe COVID-19 than firefighters (1.2% versus 0.19% respectively)^16^. This risk could be exacerbated by the elevated ability of SARS-CoV-2 to be transmitted by asymptomatic patients. Byambasuren et al. reported a 17% asymptomatic SARS-CoV-2 infection rate of total confirmed SARS-CoV-2 infected patients in a meta-analysis of data from seven countries^17^. Additionally, previous research found that in the summer of 2020, there were approximately 4.8 undiagnosed SARS-CoV-2 infections for every reported case, totaling almost 17 million undiagnosed infections^18^. Since there are a high prevalence of viral infections in trauma population and numerous asymptomatic SARS-CoV-2 cases in the general population, more information is needed to determine if first responders and trauma center staff could be at increased risk. Therefore, knowing the prevalence of COVID-19 among trauma patients would allow first responders and healthcare staff to better assess their risk of SARS-CoV-2 infection to create effective measures to mitigate the risk, along with considerations for their patients.

This study assesses the SARS-CoV-2 seropositivity of 2,542 de-identified serum samples from trauma patients using a standardized enzyme-linked immunosorbent assay (ELISA) protocol that was previously developed for the national serosurvey, conducted May 10^th^ and July 31^st^, 2020^18, 19^. The serosurvey ELISA protocol identified IgG, IgM, and IgA antibodies for the SARS-CoV-2 spike protein and its receptor binding domain (RBD). This assay can assess SARS-CoV-2 seropositivity objectively using either IgG or IgM detected levels – for both spike and RBD expression – based on a threshold determined by pre-pandemic control samples. The goal of this study was to evaluate SARS-CoV-2 seroprevalence in trauma patients to offer insightful information on the association between SARS-CoV-2 infection and trauma, which has not been previously reported. This serological study provides an in-depth assessment of SARS-CoV-2 seropositivity in trauma patients as well as detects different anti-SARS-CoV-2 antibodies with high sensitivity and specificity for each patient sample.

## MATERIALS & METHODS

### Recombinant proteins.

The procedure for protein expression and production of the selected spike and RBD in this study has been detailed previously in an established and available protocol^20, 21^. Briefly, recombinant proteins from optimized DNA constructs (Addgene #166010 for Spike, Addgene #166019 for RBD) were produced in an Expi293F mammalian expression system (Thermo Fisher Scientific). After 96 hours (Spike protein) or 72 hours (RBD protein) post-transfection, supernatants from transfected cells were harvested by centrifugation, clarified, and subjected to tangential flow filtration (TFF) prior to purification using immobilized metal affinity chromatography (IMAC). Spike proteins were desalted, and RBD proteins were further purified by size exclusion chromatography. Specific details of protein production are described by Esposito et al.^20, 21^. Final proteins were analyzed and quality-checked by SDS-PAGE with Coomassie-staining, analytical size-exclusion chromatography, and mass spectrometry. Final purified proteins were aliquoted, flash frozen in liquid nitrogen, and stored at −80°C.

### Study Design & Sample Collection.

Between April to October 2020, a total of 2,542 human serum samples were obtained as part of an ongoing National Highway Traffic Safety Administration (NHTSA) study of drug prevalence among adult (age 18+) trauma victims who were transported by EMS due to the severity of their injuries and had a trauma team activated/alerted at selected Level-1 trauma centers^22–24^. The specimens from this convenience sample were available for research purposes from patients who were already having blood drawn as part of medical treatment at the trauma centers. The toxicological analysis study followed the NHTSA’s standard panel for drugs known to impair psychomotor skills that could affect driving safety. When possible, excess serum samples from the study were made available for the serological analyses. Samples were collected at six study sites in the United States: Baltimore, Maryland (28.13%), Jacksonville, Florida (18.37%), Worcester, Massachusetts (10.70%), Charlotte, North Carolina (19.43%), Miami, Florida (16.48%), and Iowa City, Iowa (6.88%). The study was conducted in accordance with Good Clinical Practice, the principles of the Belmont Report and HHS regulations enumerated under 45 CFR 46. The Chesapeake/Advarra Institutional Review Board served as the central IRB for five sites, and the University of Florida Institutional Review Board served as the IRB of record for the Jacksonsville, FL site. De-identified samples and other data were included in the study under IRB-approved waivers of consent and authorization. All demographic information was obtained from medical records or other secondary sources such as emergency medical services run reports and crash reports. De-identified samples were then sent to NIH for SARS-CoV-2 ELISA testing on dry ice overnight and stored at −80°C until processing.

### Sample and control preparation.

Serum samples were heated at 56 °C for 1h before use to reduce the risk from any potential residual virus in the serum. The day before running ELISA, serum samples were diluted 1:400 in blocking buffer consisting of 1xPBS + 0.05% Tween20 (PBS-T) with 5.0% Nonfat Dry Milk and can be stored in 4 °C for up to 12 hours. There were four controls in technical duplicate on each plate: blank controls used for the secondary antibody signal only, SARS-CoV-2 convalescent patient sera diluted at 1: 1000 and 1: 2500 as positive controls, and archival serum as a negative control (1:400 dilution in blocking buffer). Archival serum used as negative control were collected prior to the emergence of SARS-CoV-2.

### Enzyme-linked immunosorbent assay.

The ELISA protocol was adapted from previously established protocols^18, 19, 25, 26^. This procedure utilized a semi-automated setup (BioTek Instruments EL406 washer/dispenser/stacker). High-absorption 96-well plates (NuncMaxiSorp ELISA plates; ThermoFisher) were coated with 100 μl per well of spike (1 μg/ml) or RBD (2 μg/ml) protein suspended in 1xPBS (Gibco) and incubated overnight for at least 16 hours at 4 °C. The protein solution was removed and plates were washed with 300 μL of PBS-T (0.05% Tween 20 in 1xPBS) per well three times and blocked at room temperature for 2 hours with 100 μL per well of blocking buffer (5.0% Nonfat dry milk in PBS-T). After blocking, plates were again washed three times with 300 μL of PBS-T per well. Next, 100 μl of each sample dilution or control was added in technical duplicate into the plates and incubated for 1 hour at room temperature. After sample incubation, plates were washed three times with 300 μL of PBS-T per well. Then, goat anti-human IgA, IgM, and IgG horseradish peroxidase (HRP) secondary antibodies (ThermoFisher) were diluted at 1:4000 in blocking buffer and 100 μL of each secondary antibody solution was added to each well for 1 hour. Plates were again washed three times with PBS-T, then incubated with 100 μL of 1-Step^™^ Ultra TMB-ELISA Substrate Solution (ThermoFisher) for 10 minutes followed by 100 μL of 1 N sulfuric acid STOP Solution (ThermoFisher). Within 30 minutes after adding STOP solution, optical density (OD) was measured at 450 and 650 nm using BioTek Epoch2 plate reader. To remove background, the actual absorbance was calculated as the difference between OD at 450 nm and at 650 nm before further statistical analysis.

### Statistical analysis.

Seropositivity was defined as either IgG or IgM OD levels above their respective thresholds for both the spike and RBD expression. Using both spike and RBD expression together increased sensitivity and specificity to 100% for both IgG and IgM based on evaluation with convalescent positive and archival negative controls^18, 19^. The method to determine thresholds was detailed previously using simulations of different samples and control size to model the statistical confidence over a range of disease prevalence and assay specificity. The threshold was determined as previously reported to ensure that the lower 95% confidence limit of specificity is greater than 99%. Exact binomial methods were used to compare seroprevalence between population subgroups. Multiple comparisons were corrected for using the Bonferroni method. To evaluate the association between drug exposure (each drug separately, drug classes, and any drug positivity overall) and SARS-CoV-2 serostatus, multivariable penalized likelihood logistic regression was used, adjusting for age of the trauma patient, sex, race, ethnicity, emergency room admission month, and the admission city among individuals whose samples were tested for the presence of drugs and alcohol. Analysis was done using the “logistf” package version 1.24 in R version 4.0.4^27, 28^.

## RESULTS

### Cohort Characteristics

**Figure 1:**
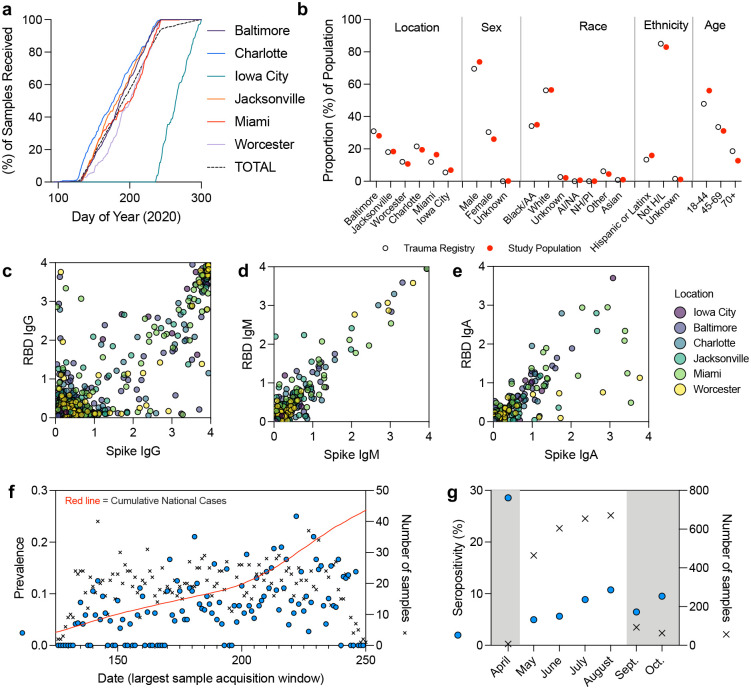
Trauma patient plasma sample collection timeline and SARS-CoV-2 serologic analysis. (a) sample collection timeline from six participating trauma centers, Baltimore (purple), Charlotte (blue), Iowa City (green), Jacksonville (red), Miami (orange), Worcester (light purple), total (dashed black). (b) Comparison of study population (red dot) to trauma registry data (open circle) within the same timeframe of collection. (c) Raw IgG serology ELISA absorbance values for SARS-CoV-2 Spike ectodomain, and receptor binding domain (RBD), (d) IgM, (e) IgA. (f) Number of samples collected (black x) versus daily seroprevalence (blue circle, see statistical methods) in the context of overall US national case trends (red line) during the main collection window. (g) Monthly seropositivity of samples, main collection window in white.

The study consists of 1434 participants identified as White (56.41%), 891 as Black or African American (35.05%), 24 as Asian or Asian American (0.94%,) 17 as Native American or Alaska Native (0.67%), 1 as Native Hawaiian or Pacific Islander (0.04%), and 175 as another race or undisclosed (6.88%). Of the study participants, four hundred and eight were identified as Hispanic or Latino (16.05%), while 2106 were identified as Not Hispanic or Latino (82.85%), and 28 unknown (1.10%). The median age was 41, with 54.64 % of participants between the ages of 18 and 44, 32.61% between the ages of 45 and 69, and 12.71% ages 70 or older. Most patients were male (72.80%) as opposed to female (26.04%). When compared to the trauma statistics generated from total admissions recorded in the trauma registry at each of the six sites during the same collection time period, the sample demographics were representative of the overall trauma population within these study sites (Fig. 1b). When compared to the general US population this trauma population is in general younger and contains more males and more non-white study participants, though this population is specific to the service areas within the bounds of the six trauma centers. Within the sample populations we were able to identify antibodies against the SARS-CoV-2 full spike ectodomain (spike) and spike receptor binding domain (RBD) with IgG, IgM and IgA classes (Fig. 1c–e). Daily measurements of seropositivity and samples collected are displayed in Fig. 1f, with monthly estimates in Fig. 1g.

### Anti-SARS-CoV-2 isotype profile among seropositive participants

**Figure 2:**
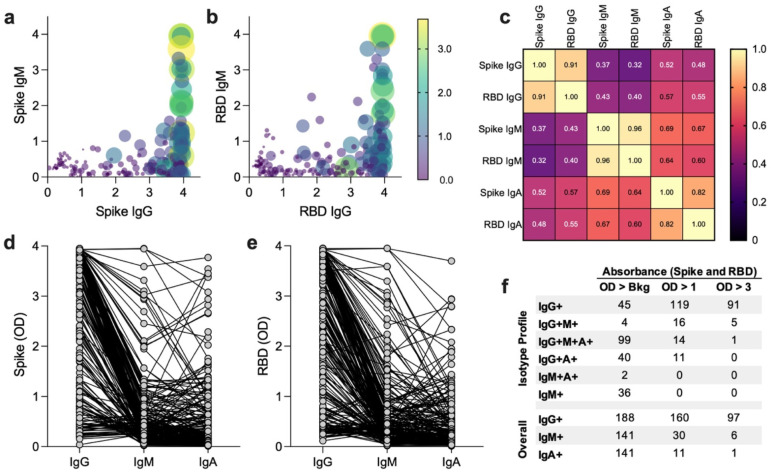
SARS-CoV-2 antibody isotype profile in seropositive patients. (a) Absorbance values for spike IgG (x-axis), IgM (y-axis), and IgA (point size/color). (b) Absorbance values for RBD IgG (x-axis), IgM (y-axis), and IgA (point size/color). (c) Correlation of expression between different serologic analytes. (d-e) Individual antibody comparing OD levels of IgG, IgM, and IgA isotypes for (d) spike and (e) RBD analytes. (f) Intensity of ELISA reading with “Bkg” = Threshold/Background, OD >1 being mid to high antibody concentration, and OD > 3 representing high and off-scale high antibody concentrations.

A range of different antibody isotype profiles were detected against both full spike ectodomain (spike, Fig. 2a) and spike receptor binding domain (RBD, Fig. 2b) antigens. A positive correlation (p ≥ 0.000001) was found with all isotypes tested, with the strongest correlations between IgG and IgA isotypes and lower correlation of IgG with IgM (Fig. 2c). The majority of those who tested positive were IgG positive (IgG+, Fig 2. d–f, n = 188/226 or 83.19%). Of those that were IgG+, more than half (51.60%) had high concentrations of antibody in their serum as measured by an OD reading greater than three, which correlates with a monoclonal recombinant anti-RBD human IgG antibody concentration of > 150 ug/ml^17^. IgM and IgA overall had lower concentrations of antibody in comparison to IgG, in agreement with previous findings in the literature.

### SARS-CoV-2 Seroprevalence in Trauma Patients from Six Sites by Demographic Groupings

**Figure 3:**
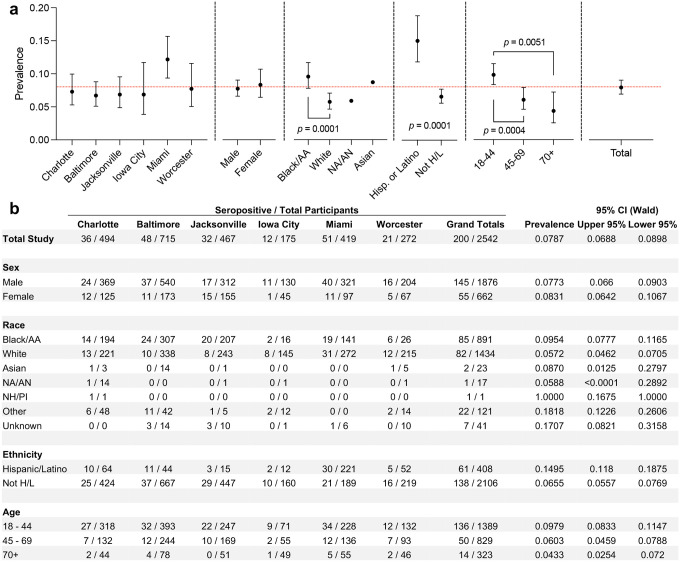
Seroprevalence of SARS-CoV-2 in Trauma Patients During the Summer 2020 COVID-19 wave. (a) Seroprevalence of SARS-CoV-2 antibodies by demographics, data are means ± 95% confidence interval (Wald), CI’s not shown for large-error (small n) samples (NA/AN, Asian). Significance = students T-test, determined by Bonferroni post-hoc adjustment for multiple comparisons. Red line = total seroprevalence of overall study population. (b) Chart of raw seropositivity data from different demographic groupings within different sites. AA = African American; NA/AN = Native American/Alaska Native; H/L = Hispanic or Latino; NH/PI = Native Hawaiian/Pacific Islander.

Overall, 7.87% (95% CI: 6.88 – 8.98) of participants were seropositive (Fig. 3). The highest seroprevalence point estimate was in Miami (12.17%, 9.36 – 15.67). Male and female participants had similar seroprevalence (male: 7.73%, 6.60 – 9.03; female: 8.31%, 6.42 – 10.67). Black/African American participants had the highest seropositivity of any race (9.54%, 7.77 – 11.65) which is significantly higher than the overall estimate (Bonferroni adjusted *p* <0.05), and significantly higher (p = 0.0001) than White participants (5.72%, 4.62 – 7.05). In addition, Hispanic/Latino participants (14.95%, 11.8 – 18.75) had the highest seroprevalence point estimate of any demographic group, which was significantly higher (p = 0.0001) than non-Hispanic/Latino participants (6.55%, 5.57 – 7.69). The youngest age group, 18 – 44 years, had significantly higher seroprevalence (9.79%, 8.33 – 11.47) than both older groups: 45 – 69 years (6.03%, 4.59 – 7.88, p = 0.0004) and 70+ years (4.33%, 2.54 – 7.20, p = 0.0051), respectively.

Of these participants, 1,679 were tested by PCR for active SARS-CoV-2 infection on site at the trauma centers. Testing approaches and rates varied by site due to differences in the availability of testing materials at the trauma centers. Of those tested for active infections, 71 patients (4.23%, 3.36 – 5.31) were positive for SARS-CoV-2. Within this group of 71 identified active infections, 41 cases were seropositive (seropositivity of participants with positive COVID test: 57.75%, 46.14 – 68.55), and the other 30 cases were seronegative (42.25%). This suggests that the majority of participants that tested positive for COVID were convalescent and most likely outside of the window of when they were most infectious; however, over 40% of these participants were pre-convalescent (seronegative) suggesting early stages of disease which is associated with higher viral loads.

### Trauma characteristics and correlations with seropositivity

**Figure 4:**
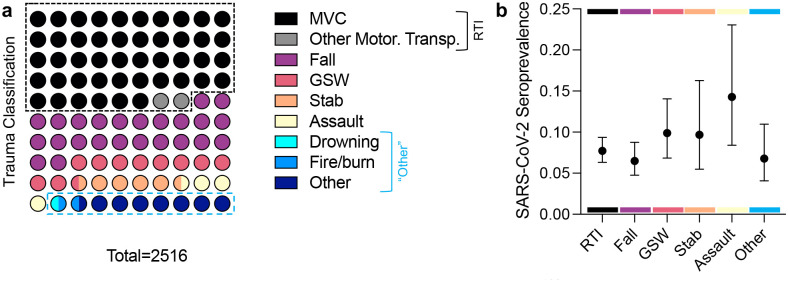
Trauma classifications and correlations with SARS-CoV-2 seropositivity. (a) Trauma classification of 2516 trauma cases. (b) SARS-CoV-2 seroprevalence in different trauma groups. Road Traffic Injuries (RTI) = Motor Vehicle crash (MVC) and Other Motorized Transportation. GSW = Gunshot Wound. Other = classified as “other” plus Drowning and Fire/Burn cases (due to low *n*). Data are point estimates ± 95% confidence interval (Wald).

The highest number of admitted trauma patients were motor vehicle crash victims (MVC’s; *n* = 1162, 46.18 %) followed by falls (*n* = 601, 23.89 %), firearm injury (GSW; *n* = 273, 10.85 %), stab (*n* = 91, 3.62 %), assault (*n* = 91, 3.62 %), drowning (*n* = 1, 0.04 %), fire/burn (*n* = 30, 1.19 %) and other traumas (*n* = 190, 7.55 %) (Fig. 4). When calculating seroprevalence point estimates, “other motorized transport injuries” were grouped with MVC’s as road traffic injuries (RTI), while drowning and fire/burn injuries were categorized as “other” (Fig. 4a). Assaults had the highest seroprevalence at 14.28% (8.4 – 23.06), though this was not significantly higher than for other trauma categories. RTIs, falls, and other traumas had similar seroprevalence (MC: 7.7%, 6.33 – 9.36; fall: 6.49%, 4.76 – 8.77; other: 6.78%, 4.08 – 10.97), while seroprevalence among firearm injuries and stab wounds was slightly higher (GSW: 9.89%, 6.84 – 14.05; stab: 9.68%, 5.49 – 16.29). Overall, type of trauma was not significantly associated with seroprevalence.

### Correlation of drug use with prior SARS-CoV-2 infection

**Figure 5:**
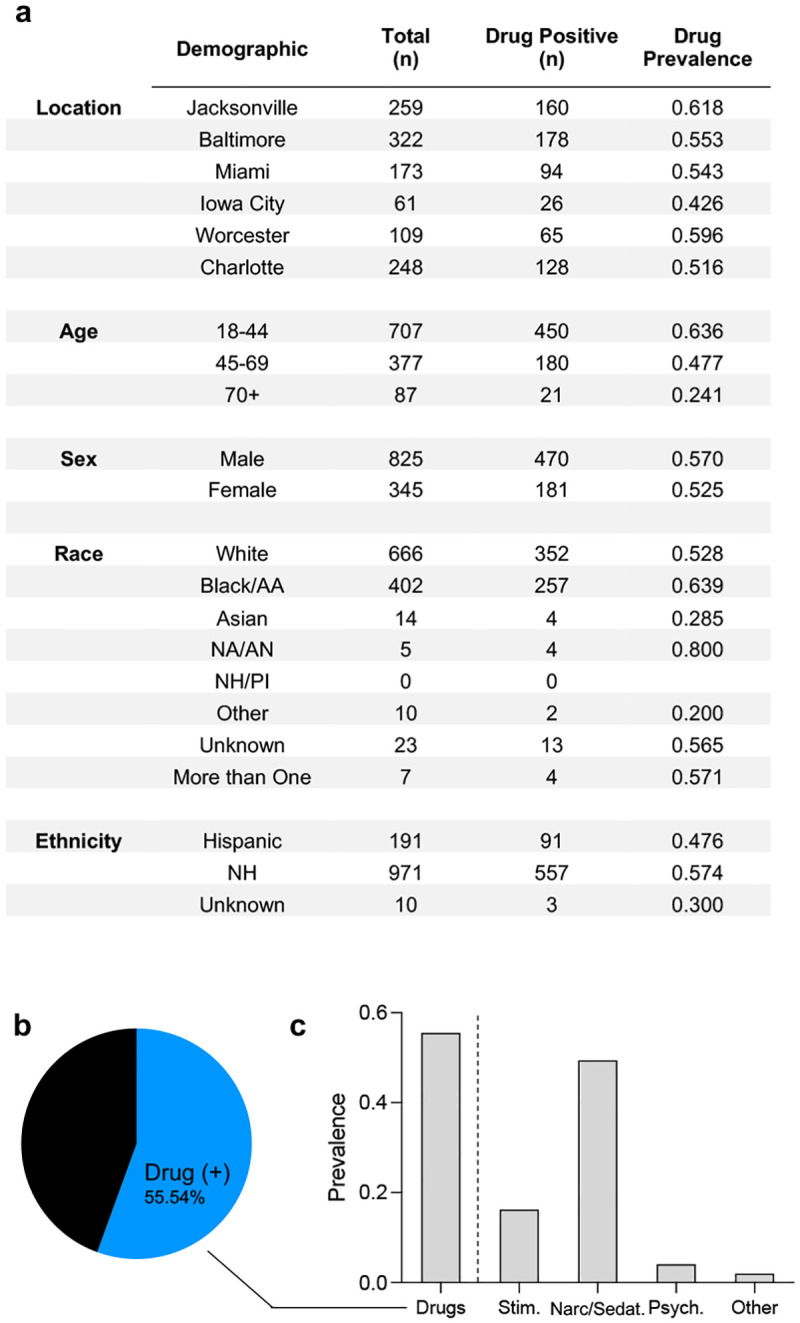
Drug prevalence in trauma patients during the summer 2020 COVID-19 wave. (a) Chart of number of trauma cases tested for drugs (motor vehicle crashes only). (b) Proportion of trauma victims that tested positive for one or more drugs in blood plasma. (c) Prevalence of different drug classifications within the trauma population^14^. Stim. = stimulants; Narc/Sedat. = Narcotic or Sedatives; Psych = Psychoactive.

Drug toxicology results, based on the NHTSA’s standard screening for drugs known to affect driving safety, were available for 1,162 of the motor vehicle crash victims included in the current seroprevalence study. Of these, 55.54 % tested positive for one or more drugs (52.69 – 58.37), including legal and decriminalized compounds such as alcohol and marijuana. The full list of drugs that were tested are available in Table 1. For further analysis, these drugs were classified in larger groupings as stimulants, narcotics or sedatives, anti-depressants, and others classification of drugs (Fig. 5).

**Figure 6:**
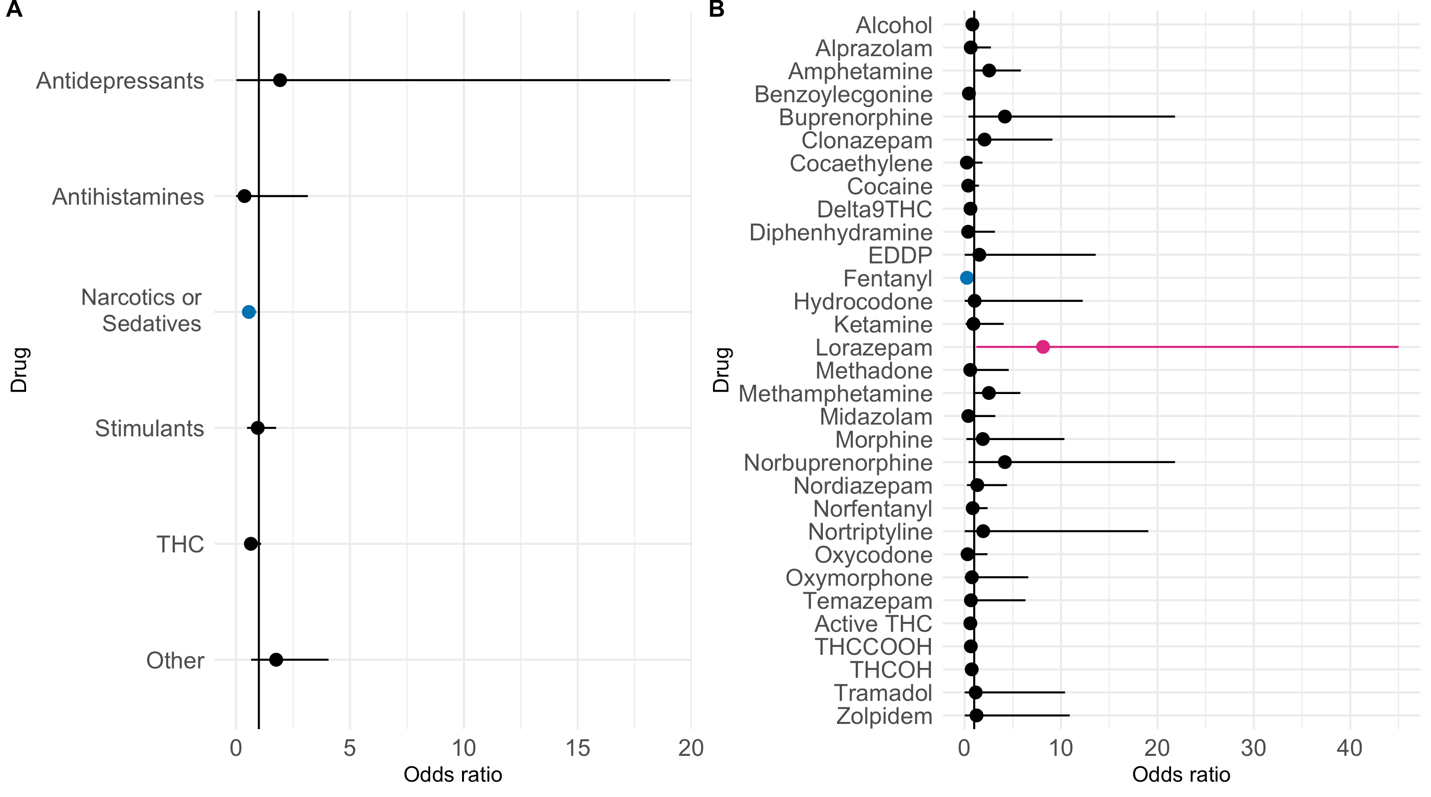
Correlation of SARS-CoV-2 Seroprevalence and Drug Presence in Trauma Patients. Odds ratios with 95% confidence intervals of a positive drug test and a positive SARS-CoV-2 seropositivity result. (a) Drug Classes. (b) Individual drugs. Red = positive correlation. Blue = Negative correlation. Black = not statistically significant.

Only two of the individual drugs tested were significantly associated with SARS-CoV-2 seropositivity after controlling for potential confounders. Samples tested positive for Lorazepam – belongs to a class of drugs known as benzodiazepines – were associated with an increased likelihood of being SARS-CoV-2 seropositive (Odds Ratio (OR): 8.14, 95% CI: 1.21 – 45.0, p = 0.03). Meanwhile, samples tested positive for fentanyl, a synthetic opioid class, were associated with a decreased likelihood of being SARS-CoV-2 seropositive (OR: 0.25, 95% CI: 0.03 – 0.95, p = 0.04). Narcotics or sedatives as a category were also negatively associated with SARS-CoV-2 seropositivity (OR: 0.56, 95% CI: 0.34 – 0.90) (Fig. 6). When comparing drug positive versus drug negative patients (those that overall had drugs detected in their toxicology), there was a slight positive trend between anti-depressant positivity and SARS-CoV-2 seropositivity, though there was no significant difference. However, samples that tested positive for narcotics or sedatives had a significantly negative correlation with SARS-CoV-2 seropositivity (p = 0.018).

## DISCUSSION

Exposure of healthcare workers and first responders to infectious diseases can be concerning for both their own health and safety, how they ensure proper care for an infected patient, as well as the health and safety of other patients. During the SARS-CoV-2 pandemic, we have witnessed the personal protective equipment shortages that can put both providers and patients at risk. One instance where control and isolation in the context of potential infectious diseases is difficult to maintain is in trauma where the main goal is to stabilize a patient’s potentially life-threatening injuries^29^. Trauma itself could also have a negative effect on the patient’s immune response against an infectious pathogen due to long-term immune dysfunction associated with traumatic injury^30^. The emergence of a novel virus such as SARS-CoV-2 gives the research community an opportunity to thoroughly characterize the potential differential burden of respiratory viruses in trauma patients at the time of admission. As such, we evaluated the prevalence of SARS-CoV-2 in trauma patients both for knowledge regarding the COVID-19 pandemic as well as data to inform the medical field of potential considerations for other respiratory viruses. In our study population of patients from urban U.S. trauma centers, we found similar and in some cases exaggerated seroprevalence compared to other seroprevalence studies in the United States^18, 31–40^. In this cohort, trauma patients who were SARS-CoV-2 seropositive were more frequently Hispanic or Black/African American, and young (< 45 years). Over 45% of seropositive trauma patients under the age of 45 identified as Black/African American, and 33% were Hispanic/Latino.

While it is difficult to compare the relative risk of SARS-CoV-2 seropositivity between the general population and the trauma population due to differences in donor recruitment and study design, we did note that in comparison to a national study conducted by our group using the same seroassays over the same time period, cities in the south/central region of the United States had higher SARS-CoV-2 prevalence in trauma patients comparison to the general population. As the samples represented this trauma population were obtained from only six trauma centers and not all Level-1 trauma facilities throughout the United States, the study cannot be used to infer seroprevalence in the overall trauma population of the United States. In addition, one of six sites, Iowa City, had a slightly delayed collection timeframe in comparison to the other five. Therefore, further investigation is necessary to understand the seroprevalence at each trauma center in comparison to its region.

Published seroprevalence estimates of the general population of Massachusetts during the same time period as this study showed a lower prevalence (4.0%) in comparison to the trauma population sampled from Worcester, MA (7.72%)^39^. Another study in Miami found that during the spring/summer of 2020 the general population had a lower seroprevalence of 6.0% compared to the trauma population at 12.17%^41^. A study in central North Carolina found increasing seroprevalence in the general community from 2.9% to 9.1% from April through October. Our estimate from Charlotte, NC which was gathered in July at 7.28%, could suggest a higher than state-average rate of SARS-CoV-2 seroprevalence in the trauma population, given the steady rate of new case diagnosis in North Carolina during this timeframe, though further analyses are needed to evaluate the probability of this phenomenon^5^.

Among motor vehicle crash victims specifically, a large proportion of trauma victims were positive for drugs or alcohol. There was a lower likelihood that these individuals had a prior SARS-CoV-2 infection if they were positive for narcotics or sedatives (including marijuana). Interestingly, there was a positive correlation with the depressant Lorazepam and SARS-CoV-2 seropositivity; this medication induces anxiolysis and sedation. In addition lorazepam can worsen obstructive pulmonary disease and lead to respiratory compromise^42^. Whether seropositivity among specific drug exposed individuals is due to chemical activity of the drug or alterations in behavior of those using these drugs remains to be determined, as do the implications of these relationships for first responders and other medical professionals needing to treat trauma patients under the influence of certain drugs. As the stimulant drug class trends higher seropositivity than THC, alcohol, benzodiazepines, or narcotics, and methamphetamine use is correlated with increased risk-taking behavior, this may explain a higher trending SARS-CoV-2 exposure. Additionally, patients using stimulants such as methamphetamine often present in the emergency room with excited delirium, spitting, and physical aggression can lead to breakdown in PPE protocols for healthcare providers.

The increased incidence of previously reported viral infections (HIV and hepatitis) in trauma victims could be co-dependent upon the increased prevalence of injectable drug use in the trauma population, creating difficulty in determining correlation versus causation^43, 44^. Given that SARS-CoV-2 is a respiratory pathogen and as of the writing of this manuscript not known to be transmitted by blood or needle sharing, this could create an important consideration for other respiratory viruses, such as influenza, necessitating an evaluation of personal protective equipment afforded to first responders, and considerations in patient care for trauma patients.

In this study, we have shown that differences in SARS-CoV-2 seroprevalence among trauma patients are, as with the general population, correlated with region, race, ethnicity, and age. There are also correlations associated with use of legal and illegal drugs, including a negative correlation of SARS-CoV-2 seropositivity with the use of narcotics or sedatives. A number of factors can affect respiratory disease spread and severity from the population level to the individual level. More densley populated areas can be subject to more rapid spread of disease due to increased likelihood of coming into contact with an infected individual^45, 46^. Unequal access to healthcare and education on disease prevention can also lead to differences in disease spread^47^. Prior to the COVID-19 outbreak, trauma patients have been shown to have higher incidence of a variety of infectious diseases, though prior research has focused on bloodborne pathogens. Respiratory diseases such as SARS-CoV-2 have the potential to complicate care plans for trauma patients who are susceptible to increased risk for post-trauma lung conditions such as pneumonia. Our results suggest a potential higher incidence of SARS-CoV-2 in trauma patients. These data are important in evaluating both the varying risks that are posed to first responders, as well as understanding potential patterns of infectious disease spread to prepare seasonal and future emerging infectious disease threats in at-risk patients.

## Figures and Tables

**Table 1:** Drugs evaluated in trauma patients and seropositivity results. Details regarding the individual drugs are displayed along with other drug name and notes regarding its application. The number detected is the number of samples that tested positive for the given drug. Number Cov2 Sero+ is the number that tested positive for IgG or IgM antibodies against SARS-CoV-2 spike protein. OTC = over the counter.

	Category 1	Category 2	Available as Prescription	Other Drug Name	Other Notes 1	Other Notes 2	Number Detected	Number Cov2 Sero+	Number Cov2 Sero−
**6-AcetylMorphine**	Narcotic or Sedative	Analgesic	YES					0	1
**7-Aminoclonazepam**	Narcotic or Sedative		YES	Klonopin	Breakdown product		3	0	3
**Acetylfentanyl**	Narcotic or Sedative	Analgesic	YES					0	1
**ActiveTHC**	Narcotic or Sedative		YES	Marijuana			324	19	305
**Alcohol**	Narcotic or Sedative	Depressant					295	20	275
**Alprazolam**	Narcotic or Sedative		YES	Xanax	Depression	Anxiety	29	1	28
**Bromazepam**	Narcotic or Sedative		YES		Pre-surgery anxiety	Anxiety	0		
**Buprenorphine**	Narcotic or Sedative	Analgesic	YES				8	1	7
**Butalbital**	Narcotic or Sedative		YES		Barbituate		4	0	4
**Carfentanil**	Narcotic or Sedative	Analgesic	YES				0		
**Carisoprodol**	Narcotic or Sedative		YES		Muscle relaxant		2	0	2
**Chlordiazepoxide**	Narcotic or Sedative		YES		Alcohol withdrawl	Anxiety	4	0	4
**Clonazepam**	Narcotic or Sedative		YES	Klonopin	Depression	Anxiety	18	1	17
**Codeine**	Narcotic or Sedative	Analgesic	YES				4	0	4
**Cyclobenzaprine**	Narcotic or Sedative		YES		Muscle relaxant		2	0	2
**Diazepam**	Narcotic or Sedative		YES	Valium	Alcohol withdrawl		16	0	16
**EDDP**	Narcotic or Sedative	Analgesic	YES	Methadone	Breakdown product		7	0	7
**Fentanyl**	Narcotic or Sedative	Analgesic	YES				73	1	72
**Fluorofentanyl**	Narcotic or Sedative	Analgesic	YES				0		
**Furanylfentanyl**	Narcotic or Sedative	Analgesic	YES				2	0	2
**Hydrocodone**	Narcotic or Sedative	Analgesic	YES				6	0	6
**Hydromorphone**	Narcotic or Sedative	Analgesic	YES				0		
**Lorazepam**	Narcotic or Sedative		YES		Seizures	Anxiety	6	2	4
**Meprobamate**	Narcotic or Sedative		YES		Anxiety		2	0	2
**Methadone**	Narcotic or Sedative	Analgesic	YES		Narcotic drug withdrawl		22	0	22
**Midazolam**	Narcotic or Sedative		YES		Pre-surgery anxiety	Anxiety	13	0	13
**Morphine**	Narcotic or Sedative	Analgesic	YES				11	1	10
**Norbuprenorphine**	Narcotic or Sedative	Analgesic	YES				8	1	7
**Nordiazepam**	Narcotic or Sedative		YES		Alcohol withdrawl	Anxiety	28	2	26
**Norfentanyl**	Narcotic or Sedative	Analgesic	YES				66	3	63
**Oxazepam**	Narcotic or Sedative		YES		Alcohol withdrawl	Anxiety	0		
**Oxycodone**	Narcotic or Sedative	Analgesic	YES				29	0	29
**Oxymorphone**	Narcotic or Sedative	Analgesic	YES				12	0	12
**Phenobarbital**	Narcotic or Sedative		YES		Barbituate	Seizures/Epilepsy	0		
**Secobarbital**	Narcotic or Sedative		YES		Barbituate		0		
**Temazepam**	Narcotic or Sedative		YES		Insomnia		7	0	7
**THCCOOH**	Narcotic or Sedative		YES	Marijuana			441	28	413
**THCOH**	Narcotic or Sedative		YES	Marijuana			189	12	177
**Tramadol**	Narcotic or Sedative	Analgesic	YES		Narcotic opioid		8	0	8
**Zolpidem**	Narcotic or Sedative		YES	Ambien	Insomnia		7	0	7
**Δ9THC**	Narcotic or Sedative		YES	Marijuana			320	19	301
**Amitriptyline**	Anti-depressant		YES		TCA		3	0	3
**Citalopram**	Anti-depressant		YES		SSRI		1	0	1
**Desipramine**	Anti-depressant		YES		TCA		0		
**Doxepin**	Anti-depressant		YES		TCA		0		
**Fluoxetine**	Anti-depressant		YES	Prozac	SSRI		0		
**Imipramine**	Anti-depressant		YES	Tofranil	TCA		0		
**Ketamine**	Anti-depressant	Psychoactive; Analgesic	YES				20	1	19
**Nortriptyline**	Anti-depressant		YES	Pamelor	TCA		6	0	6
**Sertraline**	Anti-depressant		YES		SSRI		1	0	1
**Trazadone**	Anti-depressant		YES		SSRI		0		
**Venlafaxine**	Anti-depressant		YES		SNRI		1	0	1
**α PVP**	Stimulant	Psychoactive		Bath Salts			0		
**Amphetamine**	Stimulant			Meth			58	7	51
**Benzoylecgonine**	Stimulant			Cocaine			118	4	114
**Cocaethylene**	Stimulant			Cocaine			19	0	19
**Cocaine**	Stimulant			Cocaine			45	1	44
**Ephedrine**	Stimulant		YES				0		
**MDA**	Stimulant	Psychoactive			Ecstasy-related		0		
**MDMA**	Stimulant	Psychoactive		Ecstasy			0		
**Methamphetamine**	Stimulant			Meth			58	7	51
**Methylphenidate**	Stimulant		YES	Ritalin			0		
**Phencyclidine**	Stimulant	Psychoactive		PCP			3	0	3
**Phentermine**	Stimulant		YES			Weight loss	1	0	1
**Phenylpropanolamine**	Stimulant		OTC			Weight loss	0		
**Pseudoephedrine**	Stimulant		OTC		Decongestant		0		
**Chlorpheniramine**	Antihistamine		OTC			Antihistamine	0		
**Diphenhydramine**	Antihistamine		OTC	Benadryl		Antihistamine	14	0	14
**Doxylamine**	Antihistamine		OTC	Unisom		Antihistamine	1	0	1
**Dextromethorphan**	Anti-tussive		OTC	Robutussin		Cough Medicine	4	2	2
